# Associations between online food outlet access and online food delivery service use amongst adults in the UK: a cross-sectional analysis of linked data

**DOI:** 10.1186/s12889-021-11953-9

**Published:** 2021-10-31

**Authors:** Matthew Keeble, Jean Adams, Lana Vanderlee, David Hammond, Thomas Burgoine

**Affiliations:** 1 MRC Epidemiology Unit, University of Cambridge School of Clinical Medicine, Box 285 Institute of Metabolic Science, Cambridge Biomedical Campus, Cambridge, CB2 0QQ UK; 2 École de Nutrition, Université Laval, Pavillon des Services, bureau 2729-E, 2440 boul. Hochelaga, Quebec City, QC G1V 0A6 Canada; 3 School of Public Health and Health Systems, Faculty of Health, University of Waterloo, Waterloo, ON N2L 3G1 Canada

**Keywords:** Diet, Food delivery, Food environment, Fast foods, Obesity, Online food delivery services, Public health, Takeaway foods

## Abstract

**Background:**

Online food delivery services facilitate ‘online’ access to food outlets that typically sell lenergy-dense nutrient-poor food. Greater online food outlet access might be related to the use of this purchasing format and living with excess bodyweight, however, this is not known. We aimed to investigate the association between aspects of online food outlet access and online food delivery service use, and differences according to customer sociodemographic characteristics, as well as the association between the number of food outlets accessible online and bodyweight.

**Methods:**

In 2019, we used an automated data collection method to collect data on all food outlets in the UK registered with the leading online food delivery service *Just Eat* (*n* = 33,204). We linked this with contemporaneous data on food purchasing, bodyweight, and sociodemographic information collected through the International Food Policy Study (analytic sample *n* = 3067). We used adjusted binomial logistic, linear, and multinomial logistic regression models to examine associations.

**Results:**

Adults in the UK had online access to a median of 85 food outlets (IQR: 34–181) and 85 unique types of cuisine (IQR: 64–108), and 15.1% reported online food delivery service use in the previous week. Those with the greatest number of accessible food outlets (quarter four, 182–879) had 71% greater odds of online food delivery service use (OR: 1.71; 95% CI: 1.09, 2.68) compared to those with the least (quarter one, 0–34). This pattern was evident amongst adults with a university degree (OR: 2.11; 95% CI: 1.15, 3.85), adults aged between 18 and 29 years (OR: 3.27, 95% CI: 1.59, 6.72), those living with children (OR: 1.94; 95% CI: 1.01; 3.75), and females at each level of increased exposure. We found no association between the number of unique types of cuisine accessible online and online food delivery service use, or between the number of food outlets accessible online and bodyweight.

**Conclusions:**

The number of food outlets accessible online is positively associated with online food delivery service use. Adults with the highest education, younger adults, those living with children, and females, were particularly susceptible to the greatest online food outlet access. Further research is required to investigate the possible health implications of online food delivery service use.

**Supplementary Information:**

The online version contains supplementary material available at 10.1186/s12889-021-11953-9.

## Introduction

Food prepared away-from-home is typically energy-dense and nutrient-poor [1, 2]. Over the last two decades, the number of food outlets selling such food has increased across the world [3–6]. Whilst purchasing food prepared away-from-home is likely to be determined by many factors [7], the influence of the number of food outlets physically accessible in one’s neighbourhood has been researched extensively [8–10]. Evidence from cross-sectional and longitudinal studies has shown that individuals living in areas with greater neighbourhood food outlet access consume food prepared away-from-home more frequently, and live with higher bodyweight and obesity [11, 12], and that these associations are stronger for those of lower socioeconomic position [13]. In the past, individuals may have been limited to purchasing food prepared away-from-home from outlets that were physically accessible [14]. However, changing social-norms [15], technological advances [16], widespread internet availability [17], and a desire for greater convenience [18], have influenced the emergence of alternative purchasing formats.

Online food delivery services provide online access to food outlets selling food prepared away-from-home. Based on their location, customers receive aggregated information about food outlets that will deliver to them. Customers select a food outlet and place their order through the online food delivery service platform. Orders are then forwarded to the food outlet where meals are prepared. When ready, meals are delivered by couriers who work for either the online food delivery service or independently for the food outlet. In 2018, around one in six adults in the UK had used an online food delivery service in the previous week [19]. These customers tended to have higher levels of education, were younger, male, or living with children.

Socio-ecological models propose that among other factors, an interplay between physical food outlet access and individual-level characteristics influences the purchase of food prepared away-from-home [9, 20–22]. However, little is known about the determinants of online food delivery service use. Based on findings from research investigating the role of physical food outlet access, it is reasonable to suggest that greater access to food outlets via online food delivery services is associated with more frequent use, and that the influence of this exposure varies according to customer characteristics. Food outlet characteristics may also be important. Online food delivery services facilitate access to different types of food outlets, including restaurants and takeaways, often selling a range of cuisines [23]. As taste preferences contribute to food choices [24], another possible determinant of online food delivery service use is the type of cuisine sold by accessible food outlets. Finally, food available through online food delivery services is typically energy-dense and nutrient-poor [23, 25]. Since consumption of such food has been associated with weight gain over time [26, 27], it is plausible that greater online food outlet access is associated with living with a higher bodyweight.

In this study, we investigated the association between the number of food outlets and unique types of cuisine accessible online and online food delivery service use, and in the presence of an association, whether it differed according to sociodemographic characteristics of online food delivery service customers. In secondary analyses, we investigated the association between the number of food outlets accessible online and bodyweight.

## Methods

We used Strengthening the Reporting of Observational Studies in Epidemiology (STROBE) guidelines to report the Methods of our study [28] (Checklist S1).

### Study design

In this cross-sectional analysis, we used an automated approach to collect data on the number of food outlets and unique types of cuisine accessible through an online food delivery service. We linked this with data on the use of online food delivery services and bodyweight amongst adults in the UK, collected via an online survey.

### Study population

The International Food Policy Study (IFPS) is an ongoing annual repeat cross-sectional survey conducted in Australia, Canada, Mexico, the UK, and USA. We used data collected between November–December 2019 from respondents in the UK. Data collection methods for the IFPS have been described elsewhere [29]. Briefly, respondents were recruited through Nielsen Consumer Insights Global Panel and their partners’ panels. Email invitations with links to an online survey were sent to a random sample of eligible panellists aged between 18 and 100 years. Informed consent was obtained from all respondents prior to survey completion. The IFPS received ethics clearance through a University of Waterloo Research Ethics Committee (ORE# 21460). Data collection in the UK was approved by the University of Cambridge Humanities and Social Science research ethics committee (case 19/225), and all methods were carried out in accordance with relevant guidelines and regulations.

### Online food outlet and unique type of cuisine access

The online food delivery service *Just Eat* has been available in the UK since 2006, and in 2020 was the market leader in terms of the number of food outlets registered to accept orders (around 35,000) and annual order volume (over 120 million) [30, 31]. Unlike competitors, which focus on the largest UK cities, *Just Eat* reports that it has outlets accessible across all parts of the UK [32]. Through pilot work in one region of England, we identified that 95% of food outlets registered to accept orders through a competitor (*Deliveroo*) were also registered with *Just Eat* (Additional file 1). Therefore, we used data from *Just Eat* (referred to as ‘the online food delivery service’ hereafter) as a proxy for all online food outlet access.

In November 2019, we used a web-browser extension to collect data about food outlets in England, Wales and Scotland that were accessible through the online food delivery service. First, on one weekday, we identified all food outlets registered to accept orders. Second, within 72-h, we visited the profile of each outlet on the online food delivery service website and collected data on their physical location, the types of cuisine sold (a maximum of two classifications self-determined by outlet owners), and their delivery area, which is a list of all postcode districts to which they delivered. In the UK, the first half of a full postcode is the postcode district, for example, in the *postcode* ‘CB2 0QQ’, the *postcode district* is ‘CB2’. Postcode districts are predominantly used for mail and delivery routing purposes and vary in size. Based on data from the 2011 census, the median postcode district population was 23,610 (IQR; 13,320-34,560) [33].

When completing the IFPS survey, respondents in the UK reported their residential postcode. From this, we extracted the postcode district and identified the number of food outlets accessible online by summing the number of food outlets that listed the same postcode district in their delivery area. From the accessible food outlets, we summed the number of unique types of cuisine.

### Online food delivery service use

In the IFPS survey, respondents were asked “During the past 7 days, how many meals did you get that were prepared away-from-home in places such as restaurants, fast food or takeaway places, food stands, or from vending machines?”. Respondents who had purchased at least one meal prepared away-from-home in the previous week were then asked to report the number of meals that were “Ordered using a food delivery service (e.g., Uber Eats, Just Eat, Deliveroo) and delivered”. We used answers to the second question for our primary outcome, and dichotomised respondents into those who reported use of an online food delivery service in the previous week, and those who did not.

### Body mass index and weight status

We used self-reported height and weight data for our secondary outcomes. We calculated body mass index (BMI; kg/m^2^, continuous), and used World Health Organization BMI cut-offs to classify respondents as being: ‘not overweight’ (BMI < 25 kg/m^2^); ‘overweight’ (BMI > 25–29.9 kg/m^2^); or ‘obese’ (BMI > 30 kg/m^2^). We included respondents in a ‘not available’ category when we were unable to calculate BMI due to bodyweight non-report, which is a possible reflection of social-desirability bias [34], or when calculated BMI was < 14 kg/m^2^ or > 48 kg/m^2^.

In accordance with findings from research investigating the relationship between neighbourhood food outlet access and bodyweight [12, 26], it is plausible that greater online food outlet access is positively associated with BMI and weight status. However, the focus of our study was on the association between online food outlet access and the more proximal outcome of online food delivery service use, which is a relationship less susceptible to bias from unmeasured confounding [10, 35]. Therefore, we report the findings for our secondary outcomes in additional file 2.

### Potential confounders

In the IFPS survey, respondents reported sociodemographic information. We included potential confounders, decided *a priori,* based on previous findings that they were positively associated with online food delivery service use, or purchasing food prepared away-from-home [19, 36]. Sex at birth was reported as male or female and treated as a binary variable in analysis. Age was reported in years. Due to a possible non-linear influence on food purchasing, we grouped respondents into four age categories for analysis: 18–29 years, 30–44 years, 45–60 years, > 60 years. Ethnicity was reported as the group that best described racial or ethnic background. We grouped respondents into a binary variable for analysis: ‘majority’ (white alone) or ‘minority’ (all other responses), which reflects that the majority of IFPS respondents in the UK were white. We used education and perceived income adequacy as markers of socioeconomic position [37]. Education was reported as the highest qualification completed. We categorised respondents as having: ‘low’ (high school completion or lower), ‘medium’ (some post-high school qualifications), or ‘high’ (university degree or higher) education for analysis. Perceived income adequacy was reported based on how well total monthly incomes allowed a respondent’s needs to be met. We dichotomised respondents as finding it ‘not easy’ (don’t know, refuse to answer, very difficult, difficult, or neither easy nor difficult responses), or ‘easy’ (easy or very easy responses) to make ends meet. Living with children under the age of 18 years, and smoking status in the past 30 days were reported as binary (yes/no) measures.

Food sold through online food delivery services is typically prepared in in the kitchens of existing food outlets. Therefore, online food outlet access might be a function of neighbourhood food outlet access. We used Ordnance Survey Points of Interest (OS POI) data from June 2019 to account for this. This commercial data contains information about food outlets from over 170 suppliers, is one of the most complete sources of food outlet location data available for the UK, and has been used in previous research investigating neighbourhood food outlet access [38–40]. We extracted information for the following categories as they include food outlets predominantly registered to accept orders through online food delivery services: ‘Fast food and takeaway outlets’ (food outlets serving food for consumption away from the premises), ‘Fast food delivery services’ (food outlets serving food for delivery, not through online food delivery services), ‘Fish and Chip shops’ (food outlets predominantly serving a specific type of cuisine for consumption away from the premises) and ‘Restaurants’ (food outlets serving food for consumption inside the premises) [41]. We mapped food outlets in a geographic information system (GIS) (ArcGIS version 10.7.1) using coordinates supplied in OS POI data, which have a stated accuracy of 1 m [42]. We obtained coordinates for the postcodes of IFPS respondents through Doogal (a free web-based resource), or when this was not successful, the GeoConvert tool (maintained by the UK Data Service), and mapped them in our GIS. We counted the number of food outlets listed in OS POI data within a 1600 m (1-mile) Euclidean (straight-line) radius of respondents’ postcodes to determine neighbourhood food outlet access. This distance has been shown to reflect the spatial extent of an individual’s typical shopping behaviour, and could reasonably be walked by an adult in 15–20 min [43].

### Exclusion criteria

Data were available for 4139 IFPS respondents in the UK. We removed 732 (17.7%) respondents as they had either missing postcode, covariate (except BMI and perceived income adequacy), or outcome data; lived in Northern Ireland (not covered by OS POI data); or when the total number of meals purchased away-from-home in the past 7 days exceeded 21 (as this was not considered plausible). The final analytical sample included 3067 (74.1%) respondents.

### Statistical analysis

We used Stata (version 16.1) to complete statistical analysis with a significance threshold of *p* < 0.05 throughout. To reduce non-response and selection bias, we applied post-stratification sample weights constructed based on population estimates of age, sex, ethnicity and education from the 2011 UK census [29]. We rescaled sample weights to our analytic sample, and unless specified, report weighted findings.

Residuals for all models were not normally distributed. Therefore, we modelled exposures (the number of food outlets and the number of unique types of cuisine accessible online) as quarters (Q). Q1 was the quarter with the lowest number and used as the reference category throughout statistical analyses. We used binomial logistic regression models to estimate the association between each exposure and online food delivery service use in the previous week. We completed unadjusted analyses and analyses adjusted for potential confounders of neighbourhood food outlet access, sex, age, education, perceived income adequacy, living with children and ethnicity. Where the exposure was the number of unique types of cuisine accessible online, we additionally adjusted for the number of food outlets accessible online, which was positively related.

Where the exposure was the number of food outlets accessible online, we added multiplicative interactions to our adjusted binomial logistic regression model to investigate if the association with online food delivery service use varied according to respondent education level, age and sex, and whether they lived with children. We used post-estimation Wald Tests to determine interaction significance and completed analyses stratified by the respective sociodemographic characteristic when there was evidence of a significant interaction.

### Sensitivity analyses

In sensitivity analyses, we wanted to test our assumption that we had appropriately defined neighbourhood food outlet access. We used our adjusted model for all sensitivity analyses. When constructing our variable to control for broader neighbourhood food outlet access, we first included the number of supermarkets accessible in the neighbourhood (as these outlets provide access to food sold ready-to-eat). Second, we included additional types of food outlet. Alongside the four OS POI food outlet categories initially included (‘Fast food and takeaway outlets’, ‘Fast food delivery services’ (not through online food delivery services), ‘Fish and Chip shops’ and ‘Restaurants’) we also included: ‘Cafés, snack bars and tea rooms’, ‘Convenience stores’, ‘Supermarkets’, ‘Bakeries’ and ‘Delicatessens’.

#### Secondary outcomes

We used linear regression models to investigate the association between online food outlet access and BMI, and multinomial logistic regression models to investigate the association between online food outlet access and weight status. We completed unadjusted analyses and adjusted analyses that included the aforementioned potential confounders in addition to smoking status, which is negatively related to bodyweight [44]. Due to the exploratory nature of these analyses, we did not complete sensitivity analyses or explore interactions.

## Results

### Sample characteristics

Table 1 summarises sociodemographic characteristics, access to food prepared away-from-home and online food delivery service use for the analytic sample. The median number of food outlets and unique types of cuisine accessible online was 85. Amongst respondents in the analytic sample, around one in six (15.1%) had used an online food delivery service in the previous week, the average BMI was 26.7 kg/m^2^, and 48.1% were living with overweight or obesity. Sociodemographic characteristics, access to food prepared away-from-home and food purchasing behaviours of IFPS respondents in the UK who were not included in final analyses were not materially different to those in the analytic sample (Additional file 2: Table S1 and Table S2).

**Table 1:** Characteristics of the International Food Policy Study analytic sample (*n* = 3067) ^a^

	N	%
**Sex**		
Male	1513	(49.3)
Female	1554	(50.6)
**Age**		
18–29 years	479	(15.6)
30–44 years	716	(23.4)
45–59 years	823	(26.8)
> 60 years	1048	(34.2)
**Ethnicity**		
Minority	267	(8.7)
Majority	2800	(91.3)
**Education level**		
Low	1570	(51.2)
Medium	652	(21.3)
High	845	(27.5)
**Ability to make ends meet**		
Not easy	1790	(58.4)
Easy	1277	(41.6)
**Child at home**		
No	2268	(74.0)
Yes	799	(26.0)
**Regular smoker**		
No	2417	(78.8)
Yes	650	(21.2)
**BMI**: mean (SD) ^b^	26.7	(5.3)
**Weight Status**		
Not overweight (BMI < 25 kg/m^2^)	1077	(35.1)
Overweight (BMI > 25–29.9 kg/m^2^)	877	(28.6)
Obesity (BMI > 30 kg/m^2^)	597	(19.5)
Not available	517	(16.9)
**Food exposures** (count): median (IQR)		
Online		
Outlet number	85	(34–181)
Unique cuisine type number	85	(64–108)
Neighbourhood		
Outlet number	25	(9–57)
**Online food delivery service use in the past week**		
None	2604	(84.9)
Any	464	(15.1)

^a^Data presented as weighted number of respondents (%) unless stated

^b^2551 respondents included

### Associations between online food outlet access and online food delivery service use

**Fig. 1:**
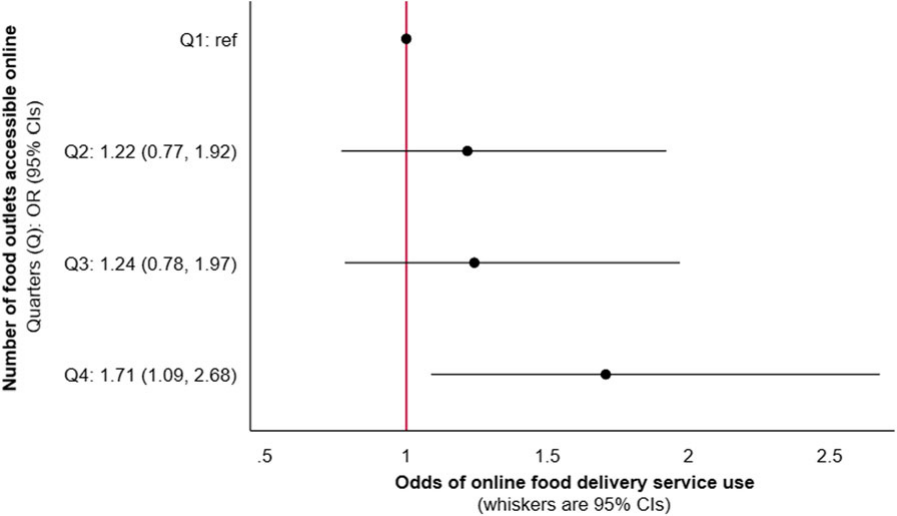
Odds of online food delivery service use in the previous week per quarter of online food outlet access amongst respondents from the International Food Policy Study (*n* = 3067). Data collected November–December 2019, modelled using adjusted binomial logistic regression. Analysis adjusted for the following potential confounders: neighbourhood food outlet access, sex, age, education level, perceived income adequacy, living with children and ethnicity. The number of food outlets accessible online for each quarter (Q) were: Q1 = 0–34, Q2 = 35–85, Q3 = 86–181, Q4 = 182–879

In our unadjusted model, the number of food outlets accessible online was positively associated with online food delivery service use in the previous week, with suggestion of a dose response relationship (Additional file 2: Table S3). In our adjusted model, associations were attenuated, however, the positive association persisted for those with the greatest online food outlet access (Fig. 1). Those with access to the greatest number of food outlets online (Q4), had 71% greater odds of using an online food delivery service in the previous week (OR: 1.71; 95% CI: 1.09, 2.68), compared to those with the lowest number (Q1).

### Interactions between online food outlet access and sociodemographic characteristics

**Fig. 2:**
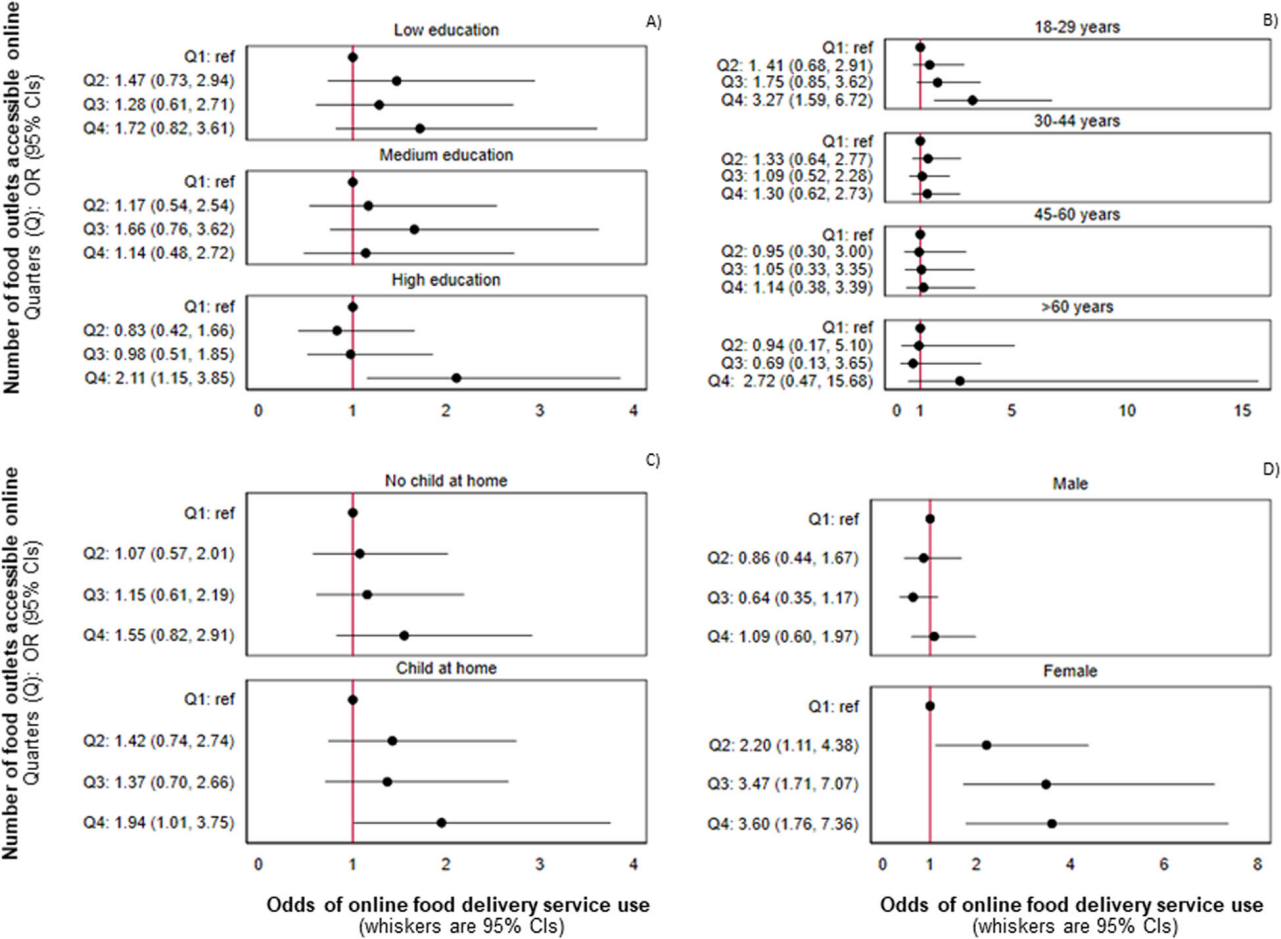
Odds of online food delivery service use in the previous week per quarter of online food outlet access amongst respondents from the International Food Policy Study (*n* = 3067), modelled using adjusted logistic regression stratified by: **A**) education level; **B**) age; **C**) living with children; and **D**) sex. Data collected November–December 2019. Note: Analyses adjusted for the following potential confounders: neighbourhood food outlet access, sex, age, education level, perceived income adequacy, living with children and ethnicity. The number of food outlets accessible online for each quarter (Q) were: Q1 = 0–34, Q2 = 35–85, Q3 = 86–181, Q4 = 182–879

There was evidence that the association between the number of food outlets accessible online and online food delivery service use in the previous week varied by sociodemographic characteristics of customers: education (*p* = 0.0015), age (*p* < 0.0001), living with children (p < 0.0001) and sex (p < 0.0001) (see Additional file 2: Table S4). Figure 2 presents findings from stratified analyses. The positive association between exposure to the greatest number of food outlets online and online food delivery service use in the previous week was evident amongst those who had the highest education (OR: 2.11; 95% CI: 1.15, 3.85), those aged between 18 and 29 years (OR: 3.27, 95% CI: 1.59, 6.72), and those living with children (OR: 1.94; 95% CI: 1.01; 3.75), but not those in other strata of these variables. The positive association increased at each level of exposure for female respondents but was entirely absent in males.

### Sensitivity analyses

In sensitivity analyses, including neighbourhood access to supermarkets and a broader range of food outlets in our adjusted models revealed similar patterns to the main analyses (Additional file 2: Table S5).

### Associations between unique types of cuisine accessible online and online food delivery service use

**Fig. 3:**
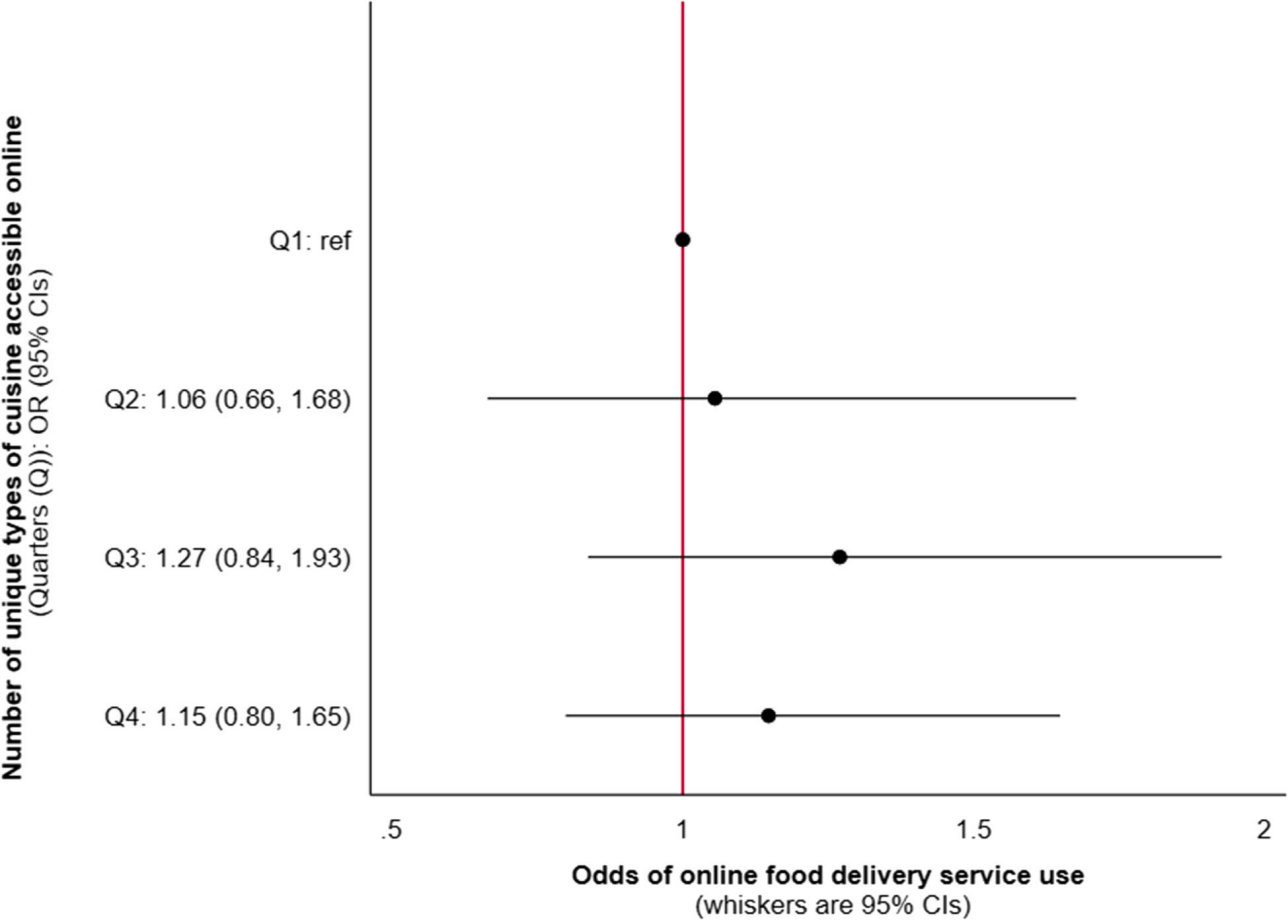
Odds of online food delivery service use in the previous week per quarter of online access to unique types of cuisine amongst respondents from the International Food Policy Study (*n* = 3067). Data collected November–December 2019, modelled using adjusted binomial logistic regression. Analysis adjusted for the following potential confounders: neighbourhood food outlet access, sex, age, education level, perceived income adequacy, living with children and ethnicity. The number of unique types of cuisine accessible online for each quarter (Q) were: Q1 = 0–64, Q2 = 65–85, Q3 = 86–108, Q4 = 109–148

In our unadjusted model, we found no evidence of an association between the number of unique types of cuisine accessible online and online food delivery service use in the previous week (Additional file 2: Table S6). Effect sizes were attenuated and remained non-significant in our adjusted model (Fig. 3).

### Secondary analyses: associations between online food outlet access and bodyweight

In our unadjusted model, the number of food outlets accessible online was inversely associated with BMI for those with the greatest access, and adults with the greatest number of food outlets accessible online had lower odds of living with obesity compared to not being overweight. These effects were attenuated to extinction in adjusted models (Additional file 2: Table S7).

## Discussion

### Summary of findings

For the first time in the international published literature, we investigated the association between multiple aspects of access to food prepared away-from-home through an online food delivery service and the use of this purchasing format. After adjustment for potential confounders, we found that adults living in the UK with the greatest number of accessible food outlets had 71% greater odds of reporting online food delivery service use in the previous week, compared to those with the fewest accessible outlets. This positive association was evident amongst those with the greatest number of accessible outlets who had the highest education, those aged between 18 and 29 years, those living with children, and females. We found no evidence of an association between the number of unique types of cuisine accessible online and online food delivery service use, or between the number of food outlets accessible online and bodyweight.

### Interpretation of findings

After adjusting for potential confounders, adults with the greatest number of food outlets accessible online had 71% greater odds of online food delivery service use in the previous week. As our study represents the first investigation into the relationship between online food outlet access and online food delivery service use, there is no existing evidence base with which to directly compare our findings. Nonetheless, socio-ecological models propose that physical exposure to food outlets in the neighbourhood can act as an environmental cue that results in purchasing food from them [9, 45], with previous research indicating that people with greater neighbourhood food outlet access purchase food prepared away-from-home more frequently [12, 46]. As food sold through online food delivery services is typically prepared in the kitchens of existing food outlets, online food outlet access may be closely linked to the number of food outlets in the neighbourhood [47]. As we controlled for this in analyses, our findings indicate that other factors cue online food delivery service use. It is plausible that seeking out this purchasing format is a response to other environmental exposures such as the visibility of food delivery couriers [48], or digital cues from targeted marketing [49], both of which are likely to be most prominent when a greater number of food outlets are accessible [50].

The positive relationship between access to the greatest number of food outlets online and use of online food delivery services in the previous week was specific to adults with the highest education, those aged between 18 and 29 years, those living with children, and females. It has previously been reported that online food delivery services are more often used by individuals with these sociodemographic characteristics [19, 51], which partly explains our findings. Younger adults and those of higher socioeconomic position spend more time using the internet [52, 53], and self-report high levels of exposure to marketing from online food delivery services [54], whilst social roles suggest that females and parents often seek out food for others [55, 56]. As a result, these groups in particular might be prone to receiving and acting on cues to visit online food delivery services. Intentionally seeking out this purchasing format, which in itself reflects shifting socio-cultural and behavioural norms regarding how food prepared away-from-home is purchased [17, 57], leads to exposure to the food outlets that are accessible online. Once this exposure occurs, access to a greater number of food outlets is likely to facilitate more choice and influence purchasing practices. In contrast, those who do not use these services are not exposed to, and thus influenced by, the number of food outlets accessible online. The decision to visit online food delivery services and to become a customer therefore appears critical, and represents an important focus for future research.

Future research might also seek to explore why we found that number of unique types of cuisine accessible online was not associated with online food delivery service use in the previous week. It is perhaps intuitive that having access to a greater number of unique types of cuisine facilitates choice and so might increase online food delivery service use [58]. However, it stands to reason that access to a greater number of unique types of cuisine leads to excessive choice [59], which might result in the decision not to use an online food delivery service to complete transactions. Furthermore, little is currently known about the purchasing process undertaken by online food delivery service customers. It might be that other food outlet characteristics that reflect determinants of purchasing food prepared away-from-home, such as food price or the availability of promotions [60–62], are more important determinants of online food delivery service use.

After adjusting for potential confounders, we observed no significant associations between the number of food outlets accessible online and either BMI or weight status. Whilst plausible, our findings of no association might also reflect that this relationship has not yet had time to develop. Although online food delivery services have been available in the UK since at least 2006, purchasing food prepared away from the home in this way has only reportedly become an established practice in more recent years [47]. A relationship may emerge in the future, especially since there is limited opportunity to purchase healthy food through these services [23]. Further research is required to understand the types and amount of food purchased through online food delivery services and how this might be associated with body weight and other customer characteristics such as education.

### Implications for policy

We have previously shown that online food delivery services are an additional purchasing format that increase overall access to food outlets selling food prepared away-from-home, but especially in areas of England with the greatest deprivation [63]. This means that whilst these services offer additional opportunities for consumption of energy-dense and nutrient-poor food amongst all populations, they may also exaggerate inequalities in the consumption of less healthy diets and related health outcomes. Despite this, in the current study, we found that people with the highest education were particularly susceptible to greater online access to food outlets (with regards to use of online food delivery services), meaning that these services may worsen diet quality in population groups who currently tend to consume the healthiest diets [64].

It is not yet clear if using online food delivery services *supplements* or *substitutes* the purchasing of food prepared away-from-home in other ways, such as directly from food outlets in person. Nonetheless, consumption of food prepared away-from-home continues to be a public health concern. To date, around half of local authorities in England have used urban planning to limit the proliferation of takeaway food outlets [65]. However, the delivery areas of food outlets registered to accept orders through online food delivery services (often takeaway food outlets) are unlikely to respect or adhere to such place-based interventions, potentially undermining them. The emergence of ‘dark-kitchens’, where food is prepared in industrial units not classified as takeaway food outlets and sold entirely through online food delivery services, are also not subject to planning policy regulation [66], and could further undermine efforts to use the planning system to improve dietary public health [67].

Alternative digital purchasing formats are available alongside online food delivery services, including independent food outlet websites and small-scale online food delivery services. However, these are less prominent and offer access to a limited number and range of food outlets. Whilst they are not comparable to internationally established online food delivery services, their emergence suggests a normalisation towards accessing and purchasing food prepared away-from-home online, which is reflected in a forecasted increase in online food delivery service use [16]. Since foods available online are typically unhealthy [25], public health policies that include online food delivery services and address online food outlet access may be required in the future. Interventions that aim to improve the nutritional quality of food prepared away-from-home are widespread but can be highly resource intensive to deliver and sustain [68–70]. However, as these interventions often include food outlets registered to accept orders through online food delivery services, they could be well-placed to help improve the nutritional quality of food accessible online. It should be noted that food available through online food delivery services does not always have an unfavourable nutrient profile [25, 71]. Implementation of public health policies with a single point of intervention (i.e. an online food delivery service) are also an opportunity to promote healthier options to customers in a consistent manner and at scale. For example, although evidence of the effectiveness of calorie menu labelling on purchasing practices is currently modest and subject to further evaluation [72], such labelling will shortly be mandatory for large chain restaurants in the UK, including on menus provided via online food delivery services [73].

Since our data were collected (November 2019), online food outlet access and use of online food delivery services has increased in many countries due to the COVID-19 pandemic [74, 75]. Greater use of these services during 2020 contributed to global revenue for online food delivery service companies exceeding $130 billion (a 27% year-on-year increase) [76], reflecting how online food delivery services have been an important means of accessing food prepared away-from-home. Moreover, urban planning reforms in England allowed new businesses, such as pubs that normally focus on serving alcohol, to offer food delivery when they had not previously [66]. This provided the opportunity for a greater number of establishments to register with online food delivery services. As a result, cues to visit these services are likely to have increased in number, contributing to greater levels of use. Long term surveillance of online food outlet access and online food delivery service use is required to understand any possible sustained public health implications.

### Strengths and weaknesses of the study

A major strength of our study was the use of automated data collection. This approach allowed unprecedented nationwide collection of exposure data, allied with contemporaneous outcome data collected from a large sample of adults. Elsewhere, use of exposure and outcome data collected at different time points is common in research investigating associations between neighbourhood food outlet access and purchasing of food prepared away-from-home [77]. This temporal mismatch, which can result in exposure misclassification, was therefore absent from our work. Moreover, the IFPS survey was developed from existing measures used in national surveys that are known to be valid and reliable [29]. And we investigated online food delivery service use as our primary outcome, which is the behaviour most proximal to online food outlet access, improving the specificity of our investigation [78, 79].

Nonetheless, our findings should be interpreted in light of some methodological limitations. Our data were from November 2019, which pre-dates changes in food purchasing practices as a result of the COVID-19 pandemic. Moreover, if food outlets were registered with the online food delivery service but not identified during data collection, our exposure measures could have been underestimated [80]. However, we identified 33,204 registered food outlets. This is similar to the “*over 30,000*” outlets reportedly signed up to the online food delivery service in contemporaneous annual reports [81], which provides confidence in the completeness of our data.

Our cross-sectional analysis prevents us from inferring a causal relationship between online food outlet access and online food delivery service use. Data on the use of online food delivery services were self-reported, which introduces the possibility of under-reporting due to social-desirability bias. However, surveys were completed online, which would have offered respondents a sense of anonymity during completion [82], and reduces this concern.

Finally, we used 1600 m straight-line buffers to define the neighbourhood of respondents. Our use of this buffer size may have influenced the number of food outlets that were deemed physically accessible as a potential confounder. Previous literature has operationalised neighbourhoods using buffers ranging from 400 m to 3200 m [83, 84]. However, 1600 m buffers have been shown to reflect the spatial extent of an individual’s typical shopping behaviour, and this distance could be reasonably walked by an adult in around 15–20 min [43].

## Conclusions

More frequent online food delivery service use could increase the consumption of energy-dense and nutrient-poor food that is typically sold away-from-home, which has known implications for health. Our study is the first to investigate the association between the number of food outlets or unique types of cuisine accessible online and the use of online food delivery services. After adjusting for a range of potential confounders, adults in the UK with the greatest number of accessible food outlets had 71% greater odds of online food delivery service use in the previous week compared to those with the lowest number. This association was particularly evident in adults who were highly educated, younger, living with children or female. We did not find evidence that the number of unique types of cuisine accessible online was associated with online food delivery service use, and the number of food outlets accessible online was not associated with bodyweight. Future research might further explore reasons for using online food delivery services and the implications for public health resulting from use of this purchasing format.

## Supplementary Information

**Additional file 1.**

**Additional file 2.**

## Data Availability

The dataset for the online food delivery service generated and analysed during the current study are not publicly available but is available from the corresponding author on reasonable request. Data from the IFPS survey is available from the corresponding author on reasonable request.

## Abbreviations

BMI: Body mass index
GIS: Geographic information system
IFPS: International Food Policy Study
OS POI: Ordnance Survey Points of Interest
STROBE: Strengthening the Reporting of Observational Studies in Epidemiology

## References

[1] Jaworowska A, Toni MB, Rachel L, Catherine T, Matthew A, Leonard S, Ian GD (2014). Nutritional composition of takeaway food in the UK. Nutr Food Sci.

[2] Marty L, Evans R, Sheen F, Humphreys G, Jones A, Boyland E, et al. The energy and nutritional content of snacks sold at supermarkets and coffee shops in the UK. J Hum Nutr Diet. 2021. Published online ahead of print. 10.1111/jhn.12880 33899984

[3] Needham C, Orellana L, Allender S, Sacks G, Blake MR, Strugnell C (2020). Food retail environments in greater Melbourne 2008–2016: longitudinal snalysis of intra-city variation in density and healthiness of food outlets. Int J Environ Res Public Health.

[4] Pinho MGM, Mackenbach JD, den Braver NR, Beulens JJW, Brug J, Lakerveld J (2020). Recent changes in the Dutch foodscape: socioeconomic and urban-rural differences. Int J Behav Nutr Phys Act.

[5] Maguire ER, Burgoine T, Monsivais P (2015). Area deprivation and the food environment over time: a repeated cross-sectional study on takeaway outlet density and supermarket presence in Norfolk, UK, 1990–2008. Health Place.

[6] Hobbs M, Mackenbach JD, Wiki J, Marek L, McLeod GFH, Boden JM (2021). Investigating change in the food environment over 10 years in urban New Zealand: a longitudinal and nationwide geospatial study. Soc Sci Med.

[7] Glanz K, Sallis JF, Saelens BE, Frank LD (2005). Healthy nutrition environments: concepts and measures. Am J Health Promot.

[8] Charreire H, Casey R, Salze P, Simon C, Chaix B, Banos A, Badariotti D, Weber C, Oppert JM (2010). Measuring the food environment using geographical information systems: a methodological review. Public Health Nutr.

[9] Story M, Kaphingst KM, Robinson-O'Brien R, Glanz K (2008). Creating healthy food and eating environments: policy and environmental approaches. Annu Rev Public Health.

[10] Mackenbach JD, Nelissen KGM, Dijkstra SC, Poelman MP, Daams JG, Leijssen JB, Nicolaou M (2019). A systematic review on socioeconomic differences in the association between the food environment and dietary behaviors. Nutrients.

[11] Kant AK, Whitley MI, Graubard BI (2015). Away from home meals: associations with biomarkers of chronic disease and dietary intake in American adults, NHANES 2005-2010. Int J Obes.

[12] Burgoine T, Forouhi NG, Griffin SJ, Wareham NJ, Monsivais P (2014). Associations between exposure to takeaway food outlets, takeaway food consumption, and body weight in Cambridgeshire, UK: population based, cross sectional study. BMJ.

[13] Burgoine T, Forouhi NG, Griffin SJ, Brage S, Wareham NJ, Monsivais P (2016). Does neighborhood fast-food outlet exposure amplify inequalities in diet and obesity? A cross-sectional study. Am J Clin Nutr.

[14] Poelman MP, Thornton L, Zenk SN (2020). A cross-sectional comparison of meal delivery options in three international cities. Eur J Clin Nutr.

[15] Maimaiti M, Zhao X, Jia M, Ru Y, Zhu S (2018). How we eat determines what we become: opportunities and challenges brought by food delivery industry in a changing world in China. Eur J Clin Nutr.

[16] Stephens J, Miller H, Militello L (2020). Food delivery apps and the negative health impacts for Americans. Front Nutr.

[17] Granheim SI, Opheim E, Terragni L, Torheim LE, Thurston M (2020). Mapping the digital food environment: a scoping review protocol. BMJ Open.

[18] Halkier B (2017). Normalising convenience food?. Food Cult Soc.

[19] Keeble M, Adams J, Sacks G, Vanderlee L, White CM, Hammond D, Burgoine T (2020). Use of online food delivery services to order food prepared away-from-home and associated sociodemographic characteristics: a cross-sectional, multi-country analysis. Int J Environ Res Public Health.

[20] Turner C, Aggarwal A, Walls H, Herforth A, Drewnowski A, Coates J, Kalamatianou S, Kadiyala S (2018). Concepts and critical perspectives for food environment research: a global framework with implications for action in low- and middle-income countries. Glob Food Sec.

[21] Egger G, Swinburn B (1997). An “ecological” approach to the obesity pandemic. BMJ.

[22] Kremers SP, de Bruijn G-J, Visscher TL, van Mechelen W, de Vries NK, Brug J (2006). Environmental influences on energy balance-related behaviors: a dual-process view. Int J Behav Nutr Phys Act.

[23] Partridge SR, Gibson AA, Roy R, Malloy JA, Raeside R, Jia SS, Singleton AC, Mandoh M, Todd AR, Wang T, Halim NK, Hyun K, Redfern J (2020). Junk food on demand: a Cross-sectional analysis of the nutritional quality of popular online food delivery outlets in Australia and New Zealand. Nutrients.

[24] Aggarwal A, Rehm CD, Monsivais P, Drewnowski A (2016). Importance of taste, nutrition, cost and convenience in relation to diet quality: evidence of nutrition resilience among US adults using National Health and nutrition examination survey (NHANES) 2007-2010. Prev Med.

[25] Wang C, Korai A, Jia SS, Allman-Farinelli M, Chan V, Roy R, Raeside R, Phongsavan P, Redfern J, Gibson AA, Partridge SR (2021). Hunger for home delivery: Cross-sectional analysis of the nutritional quality of complete menus on an online food delivery platform in Australia. Nutrients.

[26] Pereira MA, Kartashov AI, Ebbeling CB, Van Horn L, Slattery ML, Jacobs DR, Ludwig DS (2005). Fast-food habits, weight gain, and insulin resistance (the CARDIA study): 15-year prospective analysis. Lancet.

[27] Wellard-Cole L, Davies A, Allman-Farinelli M. Contribution of foods prepared away from home to intakes of energy and nutrients of public health concern in adults: a systematic review. Crit Rev Food Sci Nutr. 2021:1–12. 10.1080/10408398.2021.1887075. 10.1080/10408398.2021.1887075 33596740

[28] von Elm E, Altman DG, Egger M, Pocock SJ, Gøtzsche PC, Vandenbroucke JP (2014). The strengthening the reporting of observational studies in epidemiology (STROBE) statement: guidelines for reporting observational studies. Int J Surg.

[29] International Food Policy Study Technical Report - 2019 survey (wave 3). 2020. [http://foodpolicystudy.com/METHODS/]. Accessed 29 Apr 2021.

[30] Food delivery and takeaway market in the United Kingdom (UK) - Statistics & Facts. 2020. [https://www.statista.com/topics/4679/food-delivery-and-takeaway-market-in-the-united-kingdom-uk/]. Accessed 20 Feb 2021.

[31] Just Eat Q1 2020 Trading Update. 2020. [https://corporate.takeaway.com/media/press-releases/]. Accessed 13 Apr 2021.

[32] Just Eat Takeaway.com Annual Report 2020. 2020. [https://www.justeattakeaway.com/investors/annual-reports/]. Accessed 13 Apr 2021.

[33] 2011 Census: Key statistics for England and Wales: March 2011. 2012. [https://www.ons.gov.uk/peoplepopulationandcommunity/populationandmigration/populationestimates/bulletins/2011censuskeystatisticsforenglandandwales/2012-12-11#qualifications]. Accessed 12 Feb 2021.

[34] Burke MA, Carman KG (2017). You can be too thin (but not too tall): social desirability bias in self-reports of weight and height. Econ Hum Biol.

[35] Rummo PE, Guilkey DK, Ng SW, Meyer KA, Popkin BM, Reis JP, Shikany JM, Gordon-Larsen P (2017). Does unmeasured confounding influence associations between the retail food environment and body mass index over time? The coronary artery risk development in young adults (CARDIA) study. Int J Epidemiol.

[36] Giskes K, van Lenthe F, Avendano-Pabon M, Brug J (2011). A systematic review of environmental factors and obesogenic dietary intakes among adults: are we getting closer to understanding obesogenic environments?. Obes Rev.

[37] Galobardes B, Shaw M, Lawlor DA, Lynch JW, Davey SG (2006). Indicators of socioeconomic position (part 1). J Epidemiol Community Health.

[38] Penney LT, Burgoine T, Monsivais P (2018). Relative density of away from home food establishments and food spend for 24,047 households in England: a cross-sectional study. Int J Environ Res Public Health.

[39] Burgoine T, Harrison F (2013). Comparing the accuracy of two secondary food environment data sources in the UK across socio-economic and urban/rural divides. Int J Health Geogr.

[40] Points of interest [https://www.ordnancesurvey.co.uk/business-government/products/points-of-interest]. Accessed 20 Nov 2020.

[41] PointX classification scheme [http://www.pointx.co.uk/downloads/Classification2.0.pdf]. Accessed 20 Nov 2020.

[42] Points of Interest - Technical Specification [https://www.ordnancesurvey.co.uk/documents/product-support/tech-spec/points-of-interest-technical-specification.pdf]. Accessed 20 Nov 2020.

[43] Smith G, Gidlow C, Davey R, Foster C (2010). What is my walking neighbourhood? A pilot study of English adults' definitions of their local walking neighbourhoods. Int J Behav Nutr Phys Act.

[44] Dare S, Mackay DF, Pell JP (2015). Relationship between smoking and obesity: a cross-sectional study of 499,504 middle-aged adults in the UK general population. PLoS One.

[45] Elliston KG, Ferguson SG, Schuz N, Schuz B (2017). Situational cues and momentary food environment predict everyday eating behavior in adults with overweight and obesity. Health Psychol.

[46] Bivoltsis A, Cervigni E, Trapp G, Knuiman M, Hooper P, Ambrosini GL (2018). Food environments and dietary intakes among adults: does the type of spatial exposure measurement matter? A systematic review. Int J Health Geogr.

[47] Allen J, Piecyk M, Piotrowska M. An analysis of online shopping and home delivery in the UK [https://westminsterresearch.westminster.ac.uk/item/q16z5/analysis-of-online-shopping-and-home-delivery-in-the-uk]. Accessed 29 Apr 2021.

[48] van Rongen S, Poelman MP, Thornton L, Abbott G, Lu M, Kamphuis CBM, Verkooijen K, de Vet E (2020). Neighbourhood fast food exposure and consumption: the mediating role of neighbourhood social norms. Int J Behav Nutr Phys Act.

[49] Boelsen-Robinson T, Backholer K, Peeters A (2015). Digital marketing of unhealthy foods to Australian children and adolescents. Health Promot Int.

[50] Brown JA, Ferdinands AR, Prowse R, Reynard D, Raine KD, Nykiforuk CIJ (2021). Seeing the food swamp for the weeds: moving beyond food retail mix in evaluating young People’s food environments. SSM Popul Health.

[51] Dana LM, Hart E, McAleese A, Bastable A, Pettigrew S. Factors associated with ordering food via online meal ordering services. Public Health Nutr. 2021:1–6. 10.1017/S1368980021001294. 10.1017/S1368980021001294 PMC10195403 33762026

[52] Stamatakis E, Coombs N, Rowlands A, Shelton N, Hillsdon M (2014). Objectively-assessed and self-reported sedentary time in relation to multiple socioeconomic status indicators among adults in England: a cross-sectional study. BMJ Open.

[53] Van Volkom M, Stapley JC, Amaturo V (2014). Revisiting the digital divide: generational differences in technology use in everyday life. N Am J Psychol.

[54] Yau A, Adams J, Boyland EJ, Burgoine T, Cornelsen L, de Vocht F, Egan M, Er V, Lake AA, Lock K, Mytton O, Petticrew M, Thompson C, White M, Cummins S (2021). Sociodemographic differences in self-reported exposure to high fat, salt and sugar food and drink advertising: a cross-sectional analysis of 2019 UK panel data. BMJ Open.

[55] Fulkerson JA (2018). Fast food in the diet: implications and solutions for families. Physiol Behav.

[56] Devine CM (2005). A life course perspective: understanding food choices in time, social location, and history. J Nutr Educ Behav.

[57] Li C, Mirosa M, Bremer P (2020). Review of online food delivery platforms and their impacts on sustainability. Sustainability.

[58] Johns N, Edwards JSA, Hartwell HJ (2013). Menu choice: satisfaction or overload?. J Culin Sci Technol.

[59] Scheibehenne B, Greifeneder R, Todd PM (2010). Can there ever be too many options? A Meta-analytic review of choice overload. J Consum Res.

[60] Grunseit AC, Cook AS, Conti J, Gwizd M, Allman-Farinelli M (2019). “Doing a good thing for myself”: a qualitative study of young adults’ strategies for reducing takeaway food consumption. BMC Public Health.

[61] Bugge AB (2011). Lovin' it?. Food Cult Soc.

[62] Withall J, Jago R, Cross J (2009). Families' and health professionals' perceptions of influences on diet, activity and obesity in a low-income community. Health Place.

[63] Keeble M, Adams J, Bishop TRP, Burgoine T (2021). Socioeconomic inequalities in food outlet access through an online food delivery service in England: a cross-sectional descriptive analysis. Appl Geogr.

[64] Yau A, Adams J, White M, Nicolaou M (2020). Differences in diet quality and socioeconomic patterning of diet quality across ethnic groups: cross-sectional data from the HELIUS dietary patterns study. Eur J Clin Nutr.

[65] Keeble M, Burgoine T, White M, Summerbell C, Cummins S, Adams J (2019). How does local government use the planning system to regulate hot food takeaway outlets? A census of current practice in England using document review. Health Place.

[66] Chang M, Green L, Cummins S. All change. Has COVID-19 transformed the way we need to plan for a healthier and more equitable food environment? Urban Des Int. 2020. Published online ahead of print.

[67] Cummins S, Berger N, Cornelsen L, Eling J, Er V, Greener R, et al. COVID-19: impact on the urban food retail system, diet and health inequalities in the UK. Cities Health. 2020. Published online ahead of print.

[68] Bagwell S (2014). Healthier catering initiatives in London, UK: an effective tool for encouraging healthier consumption behaviour?. Crit Public Health.

[69] Hillier-Brown F, Lloyd S, Muhammad L, Summerbell C, Goffe L, Hildred N, Adams J (2019). Feasibility and acceptability of a takeaway masterclass aimed at encouraging healthier cooking practices and menu options in takeaway food outlets. Public Health Nutr.

[70] Hillier-Brown FC, Summerbell CD, Moore HJ, Routen A, Lake AA, Adams J, White M, Araujo-Soares V, Abraham C, Adamson AJ, Brown TJ. The impact of interventions to promote healthier ready-to-eat meals (to eat in, to take away or to be delivered) sold by specific food outlets open to the general public: a systematic review. Obes Rev. 2017;18(2):227–46. 10.1111/obr.12479. 10.1111/obr.12479 PMC5244662 27899007

[71] Horta PM, JdPM S, Rocha LL, Mendes LL (2020). Digital food environment of a Brazilian metropolis: food availability and marketing strategies used by delivery apps. Public Health Nutr.

[72] Robinson E, Marty L, Jones A, White M, Smith R, Adams J (2021). Will calorie labels for food and drink served outside the home improve public health?. BMJ.

[73] Childhood obesity: a plan for action, chapter 2. 2018. [https://www.gov.uk/government/publications/childhood-obesity-a-plan-for-action-chapter-2]. Accessed 24 Apr 2021.

[74] Jia P (2020). A changed research landscape of youth's obesogenic behaviours and environments in the post-COVID-19 era. Obes Rev.

[75] Herle M, Smith AD, Bu F, Steptoe A, Fancourt D. Trajectories of eating behavior during COVID-19 lockdown: longitudinal analyses of 22,374 adults. Clin Nutr ESPEN. 2021. Published online ahead of print. 10.1016/j.clnesp.2021.01.046 PMC7871880 33745572

[76] Jia SS, Raeside R, Redfern J, Gibson AA, Singleton A, Partridge SR (2021). #SupportLocal: how online food delivery services leveraged the COVID-19 pandemic to promote food and beverages on Instagram. Public Health Nutr.

[77] Wilkins E, Morris M, Radley D, Griffiths C (2019). Methods of measuring associations between the retail food environment and weight status: importance of classifications and metrics. SSM Popul Health.

[78] Ni Mhurchu C, Vandevijvere S, Waterlander W, Thornton LE, Kelly B, Cameron AJ, Snowdon W, Swinburn B, INFORMAS (2013). Monitoring the availability of healthy and unhealthy foods and non-alcoholic beverages in community and consumer retail food environments globally. Obes Rev.

[79] Kestens Y, Lebel A, Chaix B, Clary C, Daniel M, Pampalon R, Theriault M, SV p S (2012). Association between activity space exposure to food establishments and individual risk of overweight. PLoS One.

[80] Elliott P, Wartenberg D (2004). Spatial epidemiology: current approaches and future challenges. Environ Health Perspect.

[81] Just Eat plc Annual Report and Accounts 2018. 2019. [https://tinyurl.com/4zk9eywj]. Accessed 28 Apr 2021.

[82] Krumpal I (2013). Determinants of social desirability bias in sensitive surveys: a literature review. Qual Quant.

[83] Wilkins E, Radley D, Morris M, Hobbs M, Christensen A, Marwa WL, Morrin A, Griffiths C (2019). A systematic review employing the GeoFERN framework to examine methods, reporting quality and associations between the retail food environment and obesity. Health Place.

[84] Christensen A, Griffiths C, Hobbs M, Gorse C, Radley D (2021). Accuracy of buffers and self-drawn neighbourhoods in representing adolescent GPS measured activity spaces: an exploratory study. Health Place.

