# Supplementary material for: Associations between online food outlet access and online food delivery service use amongst adults in the UK: a cross-sectional analysis of linked data
Source: BMC Public Health. 2021 Oct 31;21:1968. doi: 10.1186/s12889-021-11953-9 (PMC8557109; doi:10.1186/s12889-021-11953-9)
Supplement: Supplementary file 1 — Additional file 1. [file 12889_2021_11953_MOESM1_ESM.pdf]

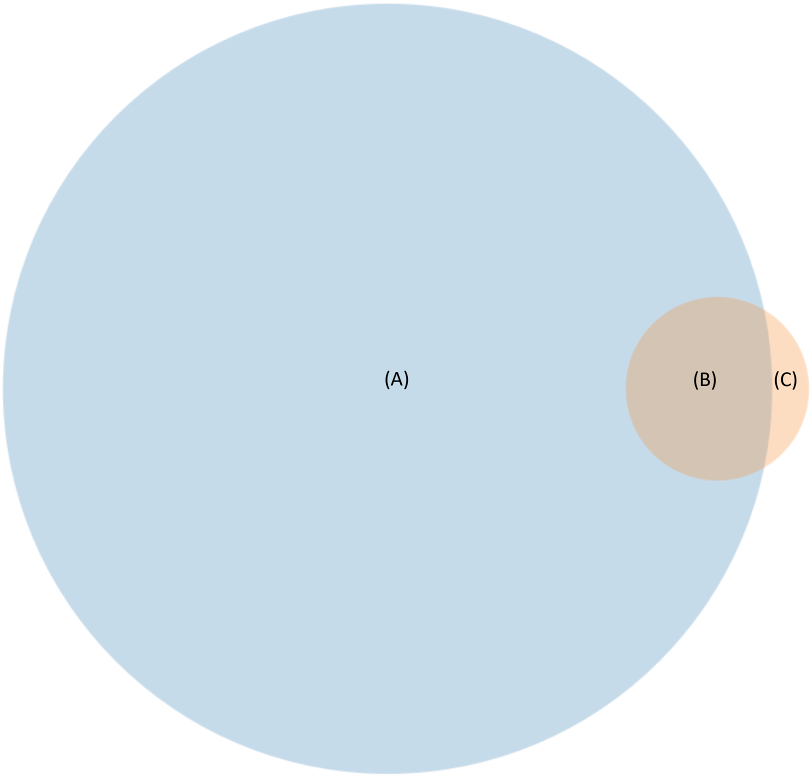

Comparison of food outlets registered with Just Eat and Deliveroo in one region in England (April 2020).

(A) = Food outlets registered to accept orders through Just Eat. N = **1673**
(B) = Food outlets registered to accept orders through Deliveroo. N = **334**
(C) = Food outlets exclusively registered to accept orders through Deliveroo. N = **91**
