# Supplementary material for: Associations between online food outlet access and online food delivery service use amongst adults in the UK: a cross-sectional analysis of linked data
Source: BMC Public Health. 2021 Oct 31;21:1968. doi: 10.1186/s12889-021-11953-9 (PMC8557109; doi:10.1186/s12889-021-11953-9)
Supplement: Supplementary file 2 — Additional file 2. [file 12889_2021_11953_MOESM2_ESM.pdf]

Table S1: Sociodemographic characteristics of UK respondents from the International Food Policy Study. Data collected November-December 2019.

	Full sample (n=4139)		Included (n=3337)		Excluded (n=732)	
**Sex**						
Male	2022	(49.0)	1648	(49.4)	341	(46.6)
Female	2117	(51.0)	1689	(50.6)	391	(53.4)
**Ethnicity**						
Minority	460	(1.0)	333	(10.0)	118	(16.2)
Majority	3648	(88.0)	2988	(89.6)	599	(81.8)
Not available	31	(11.0)	15	(0.5)	15	(2.0)
**Age**						
Mean (SD)	48.2	(16.8)	49.4	(16.9)	43.4	(15.7)
18-29 years	803	(19.4)	570	(17.1)	208	(28.4)
30-44 years	1020	(24.6)	799	(24.0)	201	(27.4)
45-59 years	1069	(25.8)	875	(26.2)	180	(24.6)
>60 years	1247	(30.1)	1093	(32.8)	143	(19.6)
**Education level**						
Low	2095	(50.6)	1688	(50.6)	364	49.6)
Medium	848	(20.5)	708	(21.2)	132	(18.1)
High	1166	(28.2)	932	(27.9)	215	(29.3)
Not available	31	(0.7)	9	(0.3)	21	(2.9)
**Ability to make ends meet**						
Not easy	2340	(56.5)	1992	(59.7)	507	69.3
Easy	1799	(43.5)	1345	(40.3)	225	30.7
**BMI** (kg/m^2): Mean (SD)	26.5 (3329 respondents)	(5.3)	26.6 (2863 respondents)	(5.3)	26.2 (457 respondents)	(5.5)
**Weight Status**						
Not overweight (BMI<25 kg/m^2)	1415	(34.2)	1170	(35.1)	227	(31.0)
Overweight (BMI$\geq$25-29.9 kg/m^2)	1097	(26.5)	945	(28.3)	136	(18.6)
Obesity (BMI$\geq$30 kg/m^2)	727	(17.6)	624	(18.7)	94	(12.9)
Not available	900	(21.7)	598	(17.9)	275	(37.5)
**Child at home**						
No	2975	(71.9)	2433	(72.9)	490	(66.7)
Yes	1158	(27.9)	901	(27.0)	239	(32.6)
Not available	6	(0.1)	3	(0.1)	3	(0.4)
**Regular smoker**						
No	3206	(77.4)	2599	(77.9)	557	(76.1)
Yes	926	(22.4)	735	(22.0)	170	(23.3)
Not available	7	(0.2)	3	(0.1)	5	(0.6)

Note: Data presented as weighted number of respondents (%) unless stated.
Full sample = all respondents*, included* = respondents with exposure data, but no covariate data, and *excluded* = respondents with no exposure data.

Table S2: Online food delivery service use in the past 7 days amongst UK respondents from the International Food Policy Study. Data collected November-December 2019.

	Full sample (n=4139)		Included (n=3337)		Excluded (n=732)	
**Online food delivery service**						
Not eligible	1185	(28.6)	902	(27.0)	213	(29.1)
Refuse to Answer	17	(0.4)	7	(0.2)	10	(13.1)
Don't Know	53	(1.3)	45	(1.3)	9	(11.8)
0	2169	(52.4)	1830	(54.9)	339	(46.3)
1+	715	(17.3)	552	(16.6)	162	(22.2)

Note: Data presented as weighted number of respondents (%), unless stated.
Full sample = all respondents*, included* = respondents with exposure data, but no covariate data, and *excluded* = respondents with no exposure data. *Not eligible* = respondents that had not purchased a meal prepared away-from-home in the past 7 days.

Table S3: Odds of online food delivery service use in the past 7 days per quarter (Q) of online food outlet access, amongst the analytic sample (n=3067). Data are from the International Food Policy Study, collected November-December 2019, modelled using unadjusted binomial logistic regression.

Food outlet number	OR	95% CI	
Q1 (0-34)	ref	-	-
Q2 (35-85)	1.35	0.91	2.03
Q3 (86-181)	1.60	1.07	2.37
Q4 (182-879)	3.11	2.17	4.45

Note: Bracketed number = number of food outlets accessible online for each Q.

Table S4: Odds ratio (OR) and 95% confidence intervals (CIs) from multiplicative interactions added to separate adjusted binomial logistic regression models. Data are from the International Food Policy Study, collected November-December 2019.

Interaction term	OR	95% CI	
**Food outlet access x education (Low = reference)**			
Q2 x Medium	0.75	0.26	2.14
Q2 x High	0.59	0.22	1.59
Q3 x Medium	1.19	0.42	3.35
Q3 x High	0.78	0.30	2.04
Q4 x Medium	0.58	0.21	1.60
Q4 x High	1.22	0.49	3.01
**Food outlet access x age (18-29 = reference)**			
Q2 x 30-44	1.05	0.38	2.90
Q2 x 45-59	0.75	0.20	2.90
Q2 x >60	0.77	0.12	5.07
Q3 x 30-44	0.71	0.26	1.93
Q3 x 45-59	0.70	0.19	2.60
Q3 x >60	0.53	0.09	2.99
Q4 x 30-44	0.68	0.27	1.70
Q4 x 45-59	0.57	0.17	1.87
Q4 x >60	1.86	0.40	8.65
**Food outlet access x sex (Male = reference)**			
Q2 x Female	3.64	1.54	8.57
Q3 x Female	4.78	2.05	11.16
Q4 x Female	3.33	1.50	7.46
**Food outlet access x child at home (No = reference)**			
Q2 x Yes	1.26	0.55	2.90
Q3 x Yes	1.01	0.44	2.29
Q4 x Yes	0.81	0.38	1.72

Note: Quarter (Q) 1 = used as reference group throughout.

Analyses adjusted for the following potential confounders: neighbourhood food outlet access, sex, age, education level, perceived income adequacy, living with children, and ethnicity. The number of food outlets accessible online for each Q were: **Q1** (0-34), **Q2** (35-85), **Q3** (86-181), **Q4** (182-879).

Table S5: Sensitivity analyses. Odds of online food delivery service use in the past 7 days per quarter (Q) of online food outlet access amongst analytic sample (n=3067) estimated using binomial logistic regression. Data are from the International Food Policy Study, collected November-December 2019.

Food outlet number	OR	95% CI	
**Neighbourhood supermarket access**			
Q1 (0-34)	ref	-	-
Q2 (35-85)	1.22	0.77	1.92
Q3 (86-181)	1.26	0.80	2.00
Q4 (182-879)	1.79	1.14	2.83
**Broader food outlet type access**			
Q1 (0-34)	ref	-	-
Q2 (35-85)	1.22	0.77	1.92
Q3 (86-181)	1.26	0.79	2.00
Q4 (182-879)	1.75	1.10	2.76

Note: Bracketed number = number of food outlets accessible online for each Q.

Broader food outlet types = nine categories included from 2019 Ordnance Survey Points of Interest data (‘Fast food and takeaway outlets’, ‘Fast food delivery services’, ‘Fish and Chip shops’, ‘Restaurants’, ‘Cafes, snack bars and tea rooms’, ‘Convenience stores’, ‘Supermarkets’, ‘Bakeries’ and ‘Delicatessens’).

Analyses adjusted for the following potential confounders: neighbourhood food outlet access, sex, age, education level, perceived income adequacy, presence of children at home, and ethnicity.

Table S6: Odds of online food delivery service use in the past 7 days per quarter (Q) of online access to unique types of cuisine, amongst the analytic sample (n=3067). Data are from the International Food Policy Study, collected November-December 2019, modelled using unadjusted binomial logistic regression.

Unique type of cuisine number	OR	95% CI	
Q1 (0-64)	ref	-	-
Q2 (65-85)	0.60	0.41	0.86
Q3 (86-108)	0.91	0.65	1.29
Q4 (109-148)	1.18	0.86	1.62

Note: Bracketed number = number unique types of cuisine accessible online for each Q.

Table S7: Secondary analyses. Associations between bodyweight and online food outlet access amongst respondents from the International Food Policy Study (n=3067). Data collected November-December 2019, modelled using multinomial linear or multinomial logistic regression.

	Model 0 [a]			Model 1 [a]		
	β.	95% CI		β.	95% CI	
**Body mass index** (kg/m^2)						
Q1 (0-34) [b]	ref	-		ref	-	-
Q2 (35-85)	-0.04	-0.74	0.67	0.03	-0.65	0.72
Q3 (86-181)	-0.17	-0.85	0.51	0.14	-0.54	0.81
Q4 (182-879)	-1.08	-1.74	-0.42	-0.11	-0.84	0.61
**Weight status** [c]	OR [d]	95% CI		OR	95% CI	
Overweight						
Q1 (0-34)	ref	-	-	ref	-	-
Q2 (35-85)	0.88	0.66	1.18	0.88	0.65	1.18
Q3 (86-181)	0.96	0.72	1.28	1.01	0.75	1.36
Q4 (182-879)	0.80	0.60	1.07	1.02	0.72	1.43
Obesity						
Q1 (0-34)	ref	-	-	ref	-	-
Q2 (35-85)	0.97	0.69	1.34	0.99	0.70	1.39
Q3 (86-181)	0.82	0.59	1.14	0.93	0.66	1.32
Q4 (182-879)	0.62	0.44	0.88	0.92	0.62	1.38
Not available [e]						
Q1 (0-34)	ref	-	-	ref	-	-
Q2 (35-85)	1.27	0.88	1.83	1.23	0.85	1.78
Q3 (86-181)	1.08	0.74	1.58	1.10	0.75	1.62
Q4 (182-879)	1.39	0.98	1.97	1.42	0.95	2.12

[a] Model 0 = unadjusted. Model 1 = adjusted for the following potential confounders: neighbourhood food outlet access, sex, age, education level, perceived income adequacy, living with children, ethnicity and smoking status.

[b] Bracketed number = number of food outlets accessible online for each quarter (Q).

[c] 'Not overweight' category used as reference group throughout.

[d] OR = Odds Ratio. CI = Confidence Intervals.

[e] Not available category = no BMI score (n=517).
